# A Novel Strain Sensor with Large Measurement Range Based on All Fiber Mach-Zehnder Interferometer

**DOI:** 10.3390/s18051549

**Published:** 2018-05-14

**Authors:** Xinran Dong, Haifeng Du, Xiaoyan Sun, Zhi Luo, Ji’an Duan

**Affiliations:** State Key Laboratory of High Performance Complex Manufacturing, College of Mechanical and Electrical Engineering, Central South University, 932 South Lushan Street, Changsha 410083, China; xrdong@csu.edu.cn (X.D.); duhaifeng@csu.edu.cn (H.D.); duanjian@csu.edu.cn (J.D.)

**Keywords:** strain sensor, photonic crystal fiber, multimode fiber, Mach-Zehnder interferometer

## Abstract

We have proposed a high sensitive photonic crystal fiber (PCF) strain sensor based on the Mach-Zehnder interferometer (MZI). The sensing head is formed by all-fiber in-line single mode-multimode-photonic-crystal-single mode fiber (SMPS) structure, using only the splicing method. Such a strain sensor exhibited a high sensitivity of −2.21 pm/με within a large measurement range of up to 5000 με and a large fringe visibility of up to 24 dB. Moreover, it was found that the strain sensitivity was weekly dependent of the length of PCF or MMF. In addition, the sensor exhibited the advantages of simplicity of fabrication, high sensitivity and larger fringe visibility.

## 1. Introduction

Optical fiber sensors have widely attracted many researchers’ attention due to its compact size, simplicity of fabrication, immunity to electromagnetic interference and high resolution [1,2]. Strain measurement plays an important role in the fields of the status and health monitoring, including aerospace, marine, civil structures, composite materials processing and so on [3,4]. To date, various strain sensors based on long period fiber gratings (LPFGs) [5], fiber Bragg gratings (FBGs) [6] and fiber in-line interferometer [7,8,9,10] have been developed rapidly, owing to their high sensitivity, ease of construction and good compatibility. Among them, various types of fiber in-line interferometric strain-sensor configurations have been demonstrated, such as Fabry-Perot interferometers based on hollow tubes [11], rectangular air bubble [3], spheroidal cavities [12] or micro-cavities fabricated by femtosecond laser [13], and Mach-Zehnder interferometers (MZIs) based on PCFs [14,15,16], multicore fiber (MCFs) [17], multimode fiber (MMF) [18,19], polymer fibers (POFs) [20], fiber taper [21] or lateral-shifted fiber splicing [22]. Particularly, PCF has unique characteristics that the ordinary optical fibers do not have, including strong birefringence, larger single-mode areas, extremely high nonlinearity and thermo-optical coefficient, owning to the exist of periodic micro-holes along fiber axis [23,24]. The low-index micro-holes is surrounded by high-index cladding that enable the total internal reflection (TIR) effect in order to guide the light into the cladding modes. In recent years, some coupler or splitter devices [23,25] and novel fiber sensors based on PCFs [8,21,26,27,28] have been attracted wide attention. For instance, in 2007, Villatoro [8] reported a temperature-insensitive strain sensor based on SMF-PCF-SMF structure. The obtained sensitivity (2.8 pm/με) is slightly higher than that of our proposed. However, the fringe visibility is about 16 dB, which is lower than ours. More importantly, the length of our proposed MZI is only 6 cm, which is about half of the MZI with SMF-PCF-SMF structure (11.9 cm). In 2015, Liu [3] reported a strain sensor based on rectangular air bubble with a high sensitivity of 43.0 pm/με. However, the strain range was too narrow, only 500 με and the air bubble was too complex to fabricate. In 2013, Khurram [16] reported a strain sensor based on twin-core photonic crystal fiber (TC-PCF) interferometer, the obtained sensitivity was only −0.31 pm/με and the fiber was high cost. In 2016, Dash [21] fabricated a MZI based on tapered PCF with an up-tapered joint. The measurement sensitivity was 1.6 pm/με. However, the tapered collapsed region could reduce the mechanical strength and cause larger lost. In 2017, Sun [29] reported a high sensitivity strain sensor by introducing higher-order interference modes using twisted MMF. The device had a high sensitivity of 42.5 pm/με. However, heating and twisting fiber enhanced the complexity of fabrication and induced the fiber strength. In a word, the MZI-based MOFs or POFs could be enhanced the cost of fibers and the MZI based on normal MMFs or PCFs need to be further improved the strain sensitivity or measurement range.

In this paper, we demonstrated a high-sensitivity strain sensor based on SMPS structure MZI, which was realized by splicing a section of MMF with PCF between two SMFs using direct fusion technology [28,30,31]. The experimental results shown that the strain sensitivity of sensor was weekly dependent of the length of MMF or PCF. The MZI sensor with 20 mm MMF, 35 mm MMF, 40 mm MMF and 50 mm MMF achieved a sensitivity of −2.21 pm/με, −1.28 pm/με, −1.36 pm/με and −1.88 pm/με, respectively. Meanwhile, the strain sensitivities of MZI sensor with 30 mm PCF, 35 mm PCF, 40 mm PCF and 45 mm PCF were −1.43 pm/με, −1.64 pm/με, −1.71 pm/με and −1.29 pm/με, respectively. In addition, the measurement range of such a strain sensor was extended up to 5000 με, while it has advantages of compact size, low cost, high fringe visibility and easy fabrication.

## 2. Fabrication of the Sensor and Sensing Principle

### 2.1. MZI Sensor Fabrication

**Figure 1:**
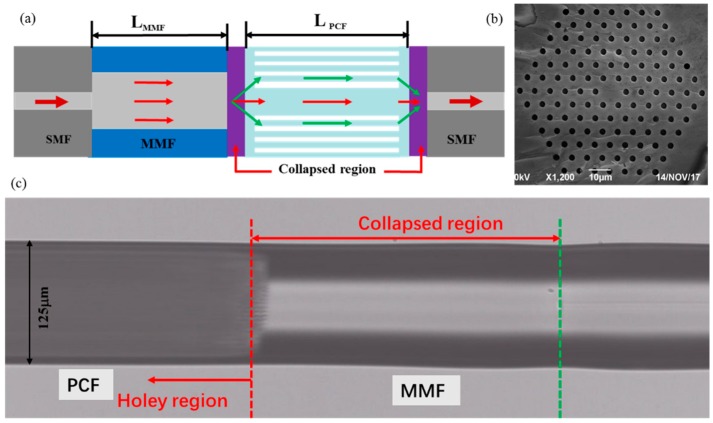
(**a**) Schematic diagram of the MZI sensor (**b**) SEM image of the cross section of PCF (**c**) Microscope of the fusion region.

Figure 1a shows the schematic diagram of the MZI sensor. A section of MMF (MMF-IRVIS-50/125 μm-25-L, OZ Optics, Carp, ON, Canada) and PCF (LMA-10, NKT Photonics, Blokken, Denmark) was spliced between two SMFs (SMF-28e, Corning, Shanghai, China) by using a fiber cleaver and a fusion splicer (FSM 80s, Fujikura, Tokyo, Japan). The cladding of fibers were removed before splicing. The core/cladding diameter of MMF and PCF employed in experiment are 50/125 μm and 10.1/125 μm, respectively. The PCF consists of six layers of air holes as shown in Figure 1b. The splicing of SMF-MMF were used standard AUTO SM Mode in the splicer menu. The optimization conditions for splicing MMF-PCF and PCF-SMF were +60 Bit arc power, 380 ms fusion time and 20 μm overlap value. Under this condition, the air holes of the PCF are slightly tapered with a length of about 91 μm and the collapsed region was formed whose overall length is approximately 240 μm as shown in Figure 1c. The fusion technology might cause some variations in that collapsed region, which will affect the obtained spectra, which is inevitable. However, the whole fabrication process uses only cleaving and splicing, which can be well controlled by a commercial fiber splicer. Therefore, the repeatability of the process is relatively good compared with the CO_2_ or femtosecond laser manufacturing. When the light was transmitted into the MMF, a part of the power could be coupled into the cladding modes of the PCF at the MMF-PCF collapsed region due to the mode filed mismatch. At the PCF-SMF collapsed region, the part light in the PCF cladding mode would couple back into the core and produce interfere with the PCF core mode. Consequently, they were coupled into the fundamental mode of the right SMF. In order to trace the interference fringes, a C+L ASE source with a wavelength of 1528~1602 nm and an optical spectrum analyzer (OSA, Agilent 86142B, wavelength range from 600 nm to 1700 nm) with a resolution of 10 pm were connected to the ends of the SMFs during the manufacturing process.

### 2.2. Sensing Principle

Theoretically, we can assume that intensity at the end of PCF stub of length L (which is known as sensing length of the device) is [26]

(1) $$I=I_{1}+I_{2}+2\sqrt{I_{1}I_{2}}\cos\frac{2\pi \Delta n_{eff}L}{\lambda }$$

where $I_{1}$ and $I_{2}$ are the intensity of the core mode and cladding mode, respectively. $\Delta n_{eff}=n_{eff}^{core}-n_{eff}^{cladding}$, which is the difference between effective refractive index of fiber core and cladding.

The m order interference valley can be written as [8,26]:

(2) $$\lambda_{m}=\frac{2\Delta n_{eff}L}{2m+1}$$

Therefore, the free spectrum range (FSR) of the interference spectrum can be expressed as [8,26]:

(3) $$FSR=\frac{\lambda^{2}}{\Delta n_{eff}L}$$

When axial strain is applied along the fiber axis, the length of MZI will be stretched, the resonant wavelength shift can be expressed using Equation (2), which is given as [8,14]:

(4) $$\Delta \lambda_{m}=\left[1+\left(\frac{L}{\Delta n_{eff}}\right)\left(\frac{\partial (\Delta n_{eff})}{\partial L}\right)\right]\lambda_{m}\varepsilon$$

It can be seen that the strain sensitivity is closely related to the change of $\Delta n_{eff}$ induced by the extended PCF length L that is $\partial (n_{eff})/\partial L$. Meanwhile, a slight physical deformation would be generated at the spliced joints when the strain ε is applied. Therefore, the out light intensity also could be changed slightly as the strain increases.

## 3. Experiment Results and Discussion

**Figure 2:**
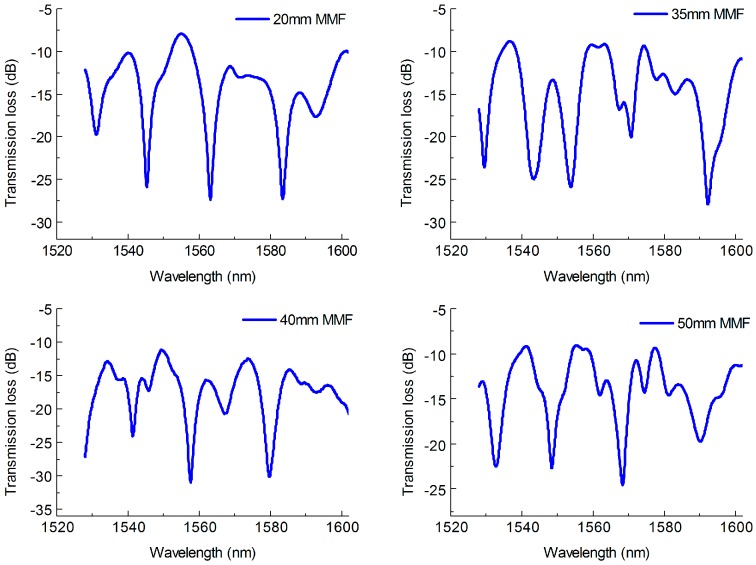
Normalized transmission spectra of the MZI with different length of MMF.

**Figure 3:**
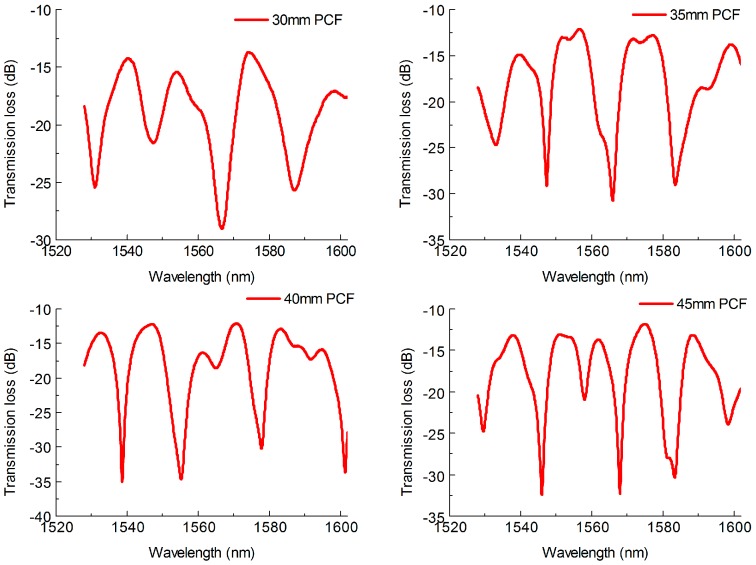
Normalized transmission spectra of the MZI with different length of PCF.

Figure 2 shows the transmission spectra of the MZI with different length of MMF. The length of PCF was set as 40 mm. It can be observed that some minor interference peaks appear as the length of MMF increases. This is mainly due to the length of MMF influencing the coupling strengths of the MMF core modes to the PCF cladding and core modes [32,33]. The multimode interference phenomenon is more obviously in the condition of longer length of MMF. In addition, the MZIs exhibited good fringe visibility, the max attenuation depth of the interference fringes for the four length of MZI were about 15 dB, 14.8 dB, 16.6 dB and 17.5 dB, respectively, as shown in Figure 2. The average values of the FSR for the corresponding MZI were about 20.7 nm, 17.1 nm, 16.6 nm and 15.9 nm, respectively, which shows a decreasing trend with the increase of the MMF length. In order to understand the influence of the PCF length to the MZI performance during our experiment, MZIs with PCF length of 30 mm, 35 mm, 40 mm and 45 mm were fabricated, and their transmission spectra are shown in Figure 3. The length of MMF was set as 30 mm. In Figure 3, it can be seen that the FSR of the MZI decrease as the length of PCF increases and the interference fringes are uniform but not look like those in Figure 2 with lots of multimode interference stripes. The average FSR of the MZI with MMF length of 30 mm, 35 mm, 40 mm and 45 mm were about 18.6 nm, 16.7 nm, 15.8 nm and 13.7 nm, respectively. Meanwhile, the MZI with different length of PCF also shown high fringe visibility, the max attenuation depth of which were about 17.2 dB, 20.3 dB, 19.9 dB and 24.1 dB, respectively, as illustrated in Figure 3. The max fringe visibility of our proposed MZI with SMPS fiber structure was higher than those of MZI with TCF-MMF-TCF structure (21 dB) [34] or MMF-TCSMF-MMF structure (14 dB) [35], MZI based on asymmetrical twin core fiber and multimode fiber (15 dB) [36] and some PCF interferometer structures, such as PCF-MZI with two waist-broadened tapers (11 dB) [37] or with up-tapered joints (11.5 dB) [38], normal PCF-MZI (21 dB) [28], TC-PCF MZI (10 dB) [16], TCF-PCF structure MZI (20 dB) [39] and S-tapered PCF (12 dB) [40]. In addition, it can be observed that the insertion loss of the proposed MZI is larger than that of MZI with SMF-PCF-SMF structure [8], which is due to the increase in the number of fusion. Moreover, the fusion parameters including arc power and arc time could be further optimized to decrease the insertion loss.

**Figure 4:**
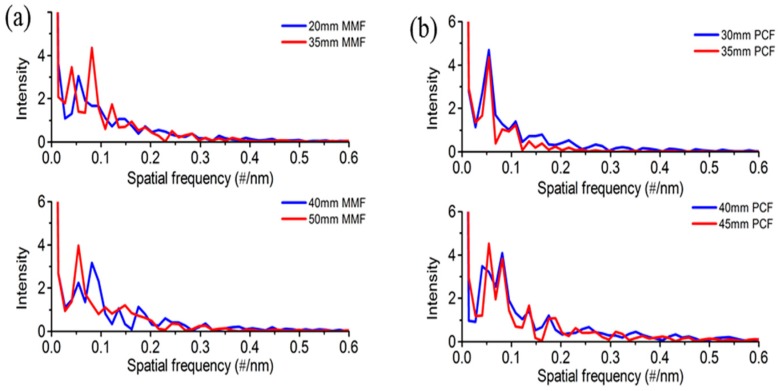
Spatial spectra of the MZI with different length of (**a**) MMF and (**b**) PCF.

In order to analyze the number and power distribution of the interference modes, the wavelength spectra in Figure 2 and Figure 3 were Fourier transformed to obtain the spatial frequency, which is shown in Figure 4. It can be seen that the dominant intensity peak at zero relates to the core modes and the intensity are primarily distributed in the core mode and the low order cladding modes. It means that the mode coupling mainly produces in the core mode and the low order cladding modes as the length of MMF or PCF varies. For the four of fabricated MZI with different length of MMF as shown in Figure 4a, it is observed that there is one dominantly excited cladding modes for the MZI with length of 20 mm, 50 mm MMF located at 0.055 #/nm and 0.054 #/nm, respectively. However, two dominant cladding modes are excited for the MZI with length of 35 mm and 40 mm MMF. It also can be found that the one dominant cladding mode for the MZI with a length 30 mm and 35 mm PCF exist in the transmission spectra while there are two dominant peaks that are excited for that with a length of 40 mm and 45 mm PCF, as shown in Figure 4b. Additionally, more higher-order cladding modes tend to be excited as the length of MMF and PCF increases. Those interferences between the core mode and the high-order cladding modes modify slightly the envelope of the main interference as shown in Figure 2 and Figure 3.

**Figure 5:**
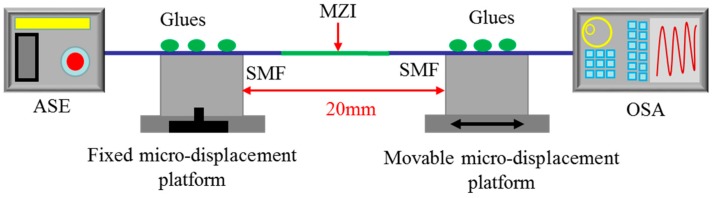
Experimental step for strain measurement.

Figure 5 shows the experimental step for strain measurement. The proposed MZI was bonded on the two micro-displacement platforms using adhesive glues. The two micro-displacement platforms (GCM-127201AM, Daheng Optics, Beijing, China) with a min displacement resolution of 10 pm and max displacement of 20 mm were applied. One platform was fixed and the other could be movable. The distance between the two platforms was set as 200 mm as shown in Figure 5. During the experiment, we moved the right micro-displacement platform forward a distance of 10 μm every time. At the same time, the OSA and ASE were connected at the two ends of SMFs to record the transmission spectra change in real time.

**Figure 6:**
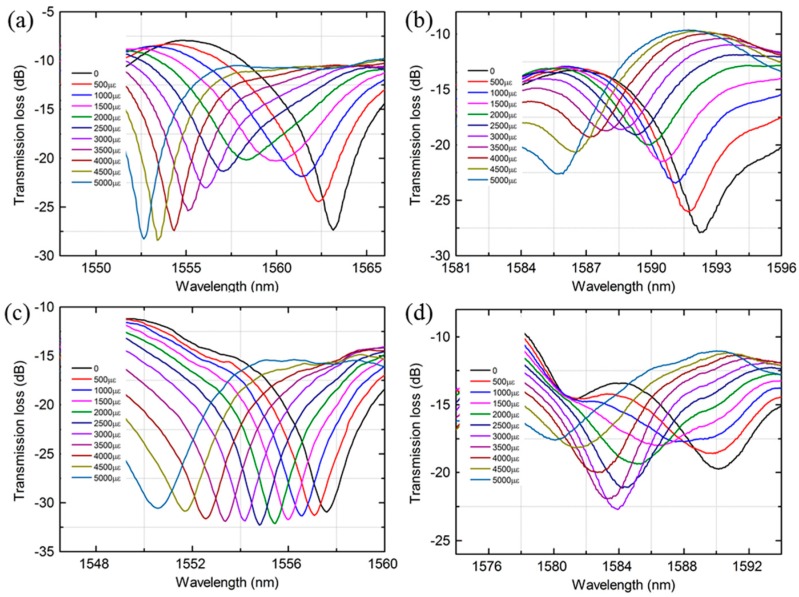
Normalized transmission spectra change of the MZI with different length of MMF as the strain increases (**a**) 20 mm (**b**) 35 mm (**c**) 40 mm (**d**) 50 mm.

**Figure 7:**
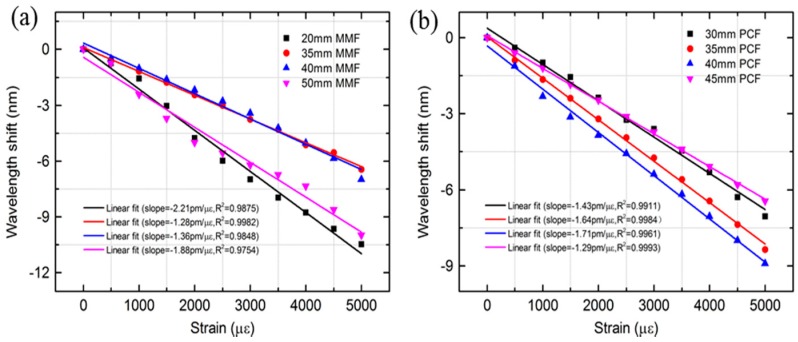
Wavelength shift of the MZI with different length of (**a**) MMF and (**b**) PCF as a function of strain.

**Figure 8:**
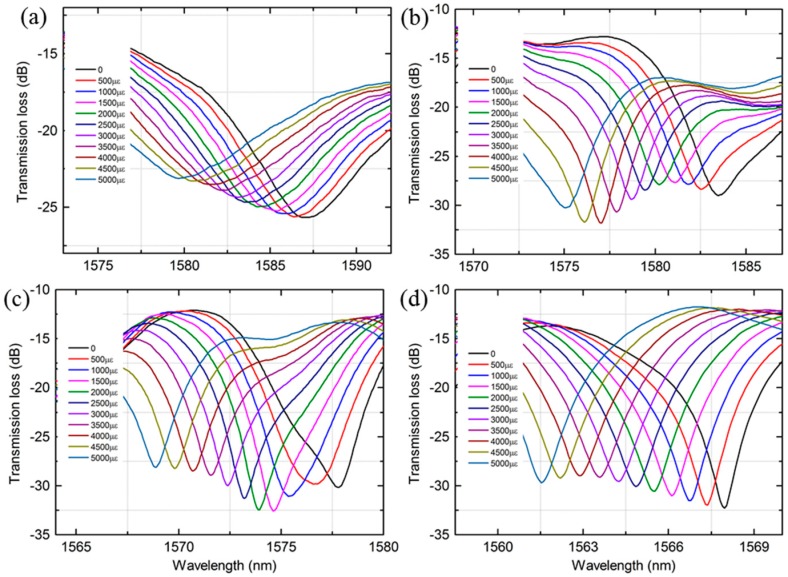
Normalized transmission spectra change of the MZI with different length of PCF as the strain increases (**a**) 30 mm (**b**) 35 mm (**c**) 40 mm (**d**) 45 mm.

Figure 6 shows the transmission spectra change of the MZI with different length of MMF as the strain increases. It can be observed that the resonant wavelengths shift monotonically towards the shorter wavelength direction when the strain increases from 0 to 5000 με. The wavelength variation of the MZI with a length of 20 mm, 35 mm, 40 mm and 50 mm MMF were 10.46 nm, 6.44 nm, 6.99 nm and 9.99 nm, respectively, as shown in Figure 6. The corresponding wavelength response to the strain exhibited good linearity, characterized by similar strain sensitivities: −2.21 pm/με, −1.28 pm/με, −1.36 pm/με and −1.88 pm/με for the four peaks, respectively, as shown in Figure 7a. Furthermore, the dependence of the strain sensitivity and the length of PCF were also investigated as illustrated in Figure 7b. The resonant wavelength of the MZI with a length of 30 mm, 35 mm, 40 mm and 45 mm PCF had a blue shift and the wavelength drift were 7.04 nm, 8.35 nm, 8.91 nm and 6.44 nm, respectively. The linear fitted plots to wavelength shift versus strain of four length of PCF are shown in Figure 7b. The strain sensitivity of the MZI with four length of PCF were −1.43 pm/με, −1.64 pm/με, −1.71 pm/με and −1.29 pm/με, respectively. The R2 of the fitted lines for the corresponding MZI were 0.9911, 0.9984, 0.9961 and 0.9993, respectively. From the above investigation, it can be seen that the length of MMF or PCF does not strong influence on the strain sensitivity. This means that strain sensitivities of the MZI are weekly depend on the length of MMF or PCF. In addition, the transmission loss of the MZI change as the strain increases is not uniform as shown in Figure 6 and Figure 8. For instance, the transmission loss of the 1563 nm dip and the 1592 nm dip were first increased slowly and then gradually decreased as shown in Figure 6a,b whereas that of the 1558 nm dip and 1587 nm dip were almost kept unchanged as shown in Figure 6c and Figure 8a, respectively. As the applied strain varies, the spliced points will be stretched along the fiber, which could cause the light intensity change in the collapsed region, the transmission loss would change as well.

In order to compare the performance of the strain sensor with previously reported ones, a comparison including strain sensitivity and measurement range is listed in Table 1. It can be seen that the strain sensitivity we measured is higher than that of LPFG fabricated by CO_2_ laser, Bragg gratings and most MZIs based on PM-PCF, Nonlinear PCF, TC-PCF and MCF as well as the tapered PCF with up-tapered joint. Meanwhile, the sensitivity we obtained can be comparable to that of modified PCF and partially filled dual-core PCF. More importantly, our sensor is easier to fabricate and has lower cost and larger measurement range. In addition, the strain sensitivity and the measurement range of SMPS structure MZI was slight higher than that of STPS structure MZI, which is we reported. However, the new structure MZI has exhibited higher fringe visibility of 24 dB and smaller size of 60 mm length than those of STPS structure MZI (20 dB, 90 mm length). Since the core diameter of MMF is larger than that of TCF, more light power will be inject into the cladding mode of PCF, leading to excite cladding modes effectively. Therefore, the new MZI has exhibited better spectra performance. The MZIs based on asymmetrical twin core fiber and MMF, S-tapered PCF, twisted MMF and microfiber fabricated by fs laser, have exhibited higher sensitivity than 4 pm/με. However, the twin core fiber is much expensive and tapered or twisted fiber could reduce the mechanical strength of fiber obviously. At the same time, the fabrication of the micro-hole using fs laser is complex and time-consuming. Those types of MZIs have a narrow measurement range of ~1400 με, which limits their application in sensing fields. The MZI with SMPS structure in our paper has many advantages of simple fabrication, larger measurement range of up to 5000 με and a high sensitivity of 2.21 pm/με as well as high fringe visibility.

**Figure 9:**
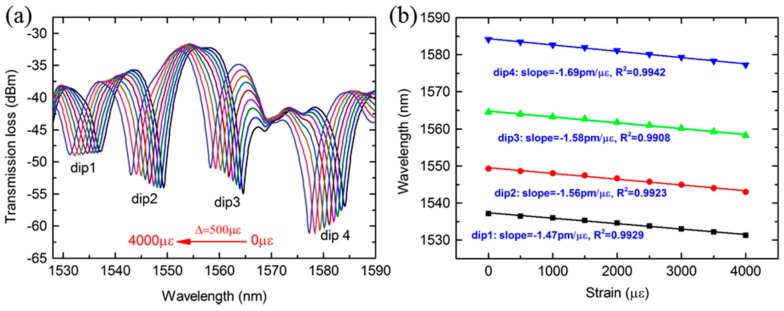
(**a**) Transmission spectra change of the MZI with SPS structure as the strain increases (**b**) Wavelength shift of the four dips as a function of strain.

Additionally, in order to compare the strain sensitivity with SMF-PCF-SMF (SPS) structure, the strain characteristics of MZI with 40 mm PCF was demonstrated experimentally, as shown in Figure 9a. It is observed that the dips have a redshift when the strain increases from 0 to 4000 με. The measured sensitivities for the dips are are −1.47 pm/με, −1.56 pm/με, −1.58 pm/με and −1.69 pm/με, respectively, as shown in Figure 9b. From the investigation, we can find that the dip with lower order of cladding mode has exhibited higher sensitivity and the sensitivity of MZI with SMPS structure (−2.21 pm/με) is obviously higher than that of MZI with SPS structure (−1.7 pm/με). If the MMF and PCF are treated as an elastic structure, when a same strain ε is applied along the fiber axial, the stress of fiber cross section of the SMPS structure is bigger than that of SPS structure according to the Hooke’s law. This means the photo-elastic effect of the SMPS structure would be stronger than that of SPS structure, so the wavelength will be more sensitive to the applied strain.

**Figure 10:**
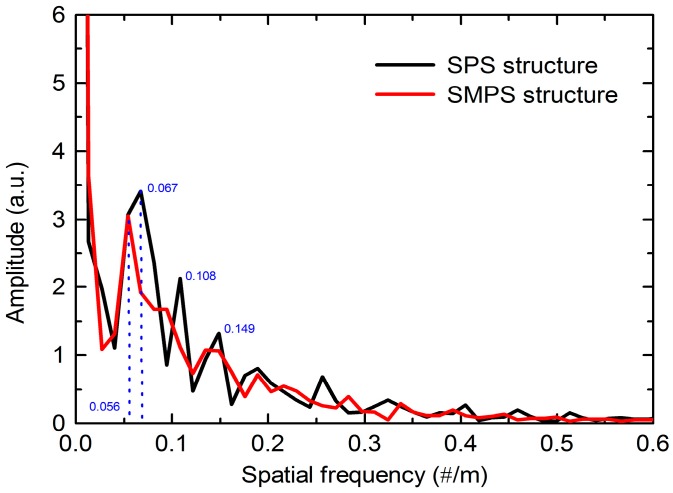
Spatial frequency of the MZI with SPS and SMPS structure.

In order to analyze the number and the power distribution of the interference modes, the experimental spectra of two structures are taken fast Fourier transform (FFT) to obtain the spatial frequency spectra, as shown in Figure 10. It can be seen that the dominant intensity peak at zero relates to the core modes and the intensity are primarily distributed in the core mode and the low order cladding modes. There are three dominant peaks in the spatial spectra for SPS structure MZI, whose dominant maximum frequency are located at 0.067#/nm, 0.108#/nm and 0.149#/nm, respectively. However, there is one dominant peak in the spatial spectra for SMPS structure MZI. The dominant maximum frequency is located at ~0.0056 #/nm. From above investigation, it can be seen that the SMPS structure excites lower order cladding modes. Therefore, the proposed MZI will present higher sensitivity according to Equations (2) and (4). In addition, the core diameter of the MMF is larger than that of SMF, that is, the mode field diameter of the MMF is much larger than SMF. Thus, the power of the light injected to the cladding mode of the PCF would be dramatically enhanced. The modes coupling for SMPS is mainly excited with the low order cladding modes as shown in Figure 10. Moreover, the cladding modes of the PCF could be excited due to the applying of MMF, which is different from the SPS structure that the core modes and cladding modes are excited through the mode field mismatch between two SMFs and PCF fiber. Those are the reasons why we choose MMF as a part of the sensor.

**Figure 11:**
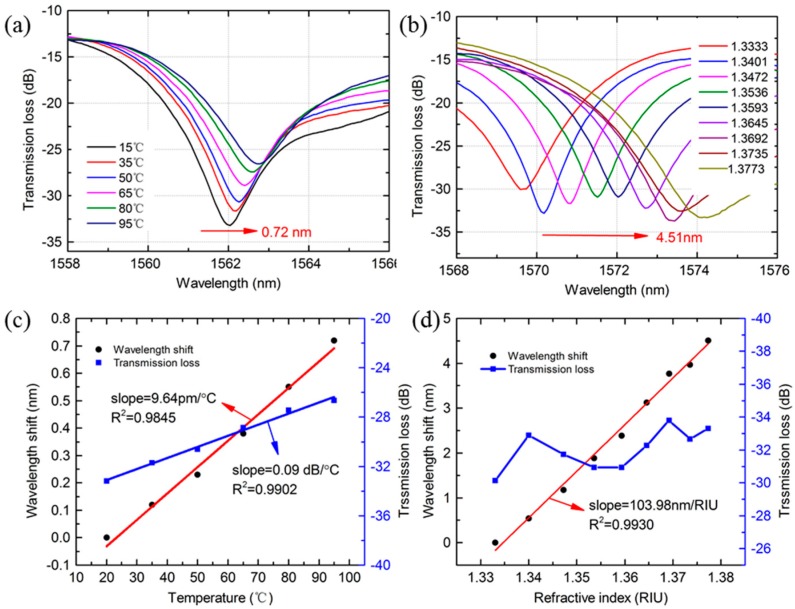
(**a**,**b**) Transmission spectra change of the MZI as the temperature or RIs change, respectively; (**c**,**d**) Wavelength shift and transmission loss of the MZI as a function of temperature or RIs, respectively. (MZI with 20 mm MMF and 40 mm PCF).

In order to know the sensitivity of the proposed MZI to temperature and surrounding refractive index, the MZI with 20 mm MMF and 40 mm PCF was chosen to be characterized. From Figure 11a,b, it can be seen that the dip has a red shift as the temperature and RI increases. The wavelength shift variations of the dip were 0.72 nm and 4.51 nm, respectively. A temperature sensitivity of 9.64 pm/°C with a linearity of 0.9845 in range of 15 °C~95 °C, and a RI sensitivity of 103.98 nm/RIU with a linearity of 0.9930 in range of 1.3333~1.3773 were obtained by linear fitting as shown in Figure 9c,d, respectively. In addition, the transmission loss of the dip was linearly increased from −33.2 dB to −26.65 dB as the temperature changed whereas the response of transmission loss to RIs change was small and nonlinear. The fitting slope of the dip was 0.09 dB/°C with a linearity of 0.9902 as the temperature increased, as shown in Figure 9c. From above investigation, it can be that the proposed MZI is insensitive to temperature, which is benefit for the application in field of strain sensing.

## 4. Conclusions

In conclusion, a high-sensitivity strain sensor based on all fiber inline MZI with SMPS structure was proposed and demonstrated experimentally. A high strain sensitivity of −2.21 pm/με is obtained in the range of 0~5000 με. The measurement range of the sensor was larger than most reported MZIs, including the normal PCF and tapered structure MZI as well as PCF interference-based PM-PCF or TC-PCF. Furthermore, the experimental results proved that the strain sensitivity of the sensor did not strongly depend on the length of MMF or PCF. The advantage of the proposed sensor was simplicity of fabrication. In addition, it exhibited large strain measurement range of up to 5000 με and high fringe visibility of ~24 dB and high sensitivity, which means that the structure is attractive for the development of strain sensors.

## Figures and Tables

**Table 1:** Performance comparison with different types of MZIs and gratings.

Structure Type	Strain Sensitivity (pm/με)	Measurement Range (με)	Reference
LPFG fabricated by CO_2_ laser	0.76	1200	[41]
FBGs	1.22	1800	[42]
Polarization-maintaining PCF (PM-PCF)	1.01	7000	[7]
Nonlinear PCF	0.93	4000	[30]
Twin-core PCF (TC-PCF)	0.31	4000	[16]
Multi-core fiber (MCF)	1.4	1600	[17]
Tapered PCF with up-tapered joint	1.6	1000	[21]
Modified PCF	1.98	1300	[43]
STPS structure MZI	1.95	4000	[39]
Partially filled dual-core PCF (DC-PCF)	2.08	1400	[44]
asymmetrical twin core fiber and MMF	4.01	1400	[36]
S-tapered PCF	4.3	800	[27]
Twisted MMF	7	1000	[29]
Microfiber fabricated by fs laser	6.8	1000	[9]
MZI with SMPS structure	2.21	5000	our paper
